# Moving from Latent to Manifest Problem: Trajectories Across Scientific and Public Salience of Invasive Alien Species

**DOI:** 10.1007/s00267-020-01404-3

**Published:** 2021-01-12

**Authors:** J. Vaas, P. P. J. Driessen, M. Giezen, F. van Laerhoven, M. J. Wassen

**Affiliations:** 1 Copernicus Institute of Sustainable Development, Utrecht University, Utrecht, The Netherlands; 2 Department of Human Geography, Planning, and International Development Studies, University of Amsterdam, Amsterdam, The Netherlands

**Keywords:** Invasive alien species, Latency, Management, Inertia, Public salience, Scientific salience

## Abstract

Who worries first about an invasive alien species: scientists or the general public, or do both become concerned simultaneously? Taking thirteen invasive alien species in the Netherlands, this article reconstructs the development of their public and scientific salience: the attention they attracted and the knowledge about them. Salience was assessed from the number of publications from 1997 onwards in the LexisNexis newspaper database and Scopus scientific database. Three trajectories were derived for a species to move from being a latent problem with low salience toward a manifest status with high public and scientific salience. In the most common trajectory, scientific salience increased first, followed by an increase in public salience. We probed the merit of this concept of trajectories by examining the action undertaken for a representative species of the trajectories. We assigned each of these three species a code for inertia and inaction based on the content of a hundred newspaper articles and all available government documents. Knowing the scientific and public salience of these species clarifies why the actions to deal with them differed even though from an ecological perspective they warranted similar attention. The typology of public and scientific salience and the problem trajectories developed in this article together offer a structured approach for understanding an invasive alien species and provide pointers for engaging a community in managing that species.

## Introduction

It is widely accepted that to deal successfully with environmental issues, it is crucial to involve communities in the management of the environment (Armitage 2009; Folke et al. 2005; Lührs et al. 2018; Papadopoulos and Warin 2007; Turnhout et al. 2010). However, involving people in the management of environmental problems is one of the major challenges of environmental governance. Despite the limited empirical evidence that involving local actors results in better outcomes of decision-making (Newig and Fritsch 2009), it is consistently claimed that higher acceptance and implementation of environmental decisions follow from participatory processes (Newig et al. 2018). For involvement to be achieved, the communities in question need to be aware of the issue and deem it to be important. One impediment to involvement of people could be their ignorance of a problem and of the risk it poses (e.g., Esteve et al. 2018; Fizer et al. 2018). Supplying them with additional knowledge might raise their awareness but does not necessarily result in them deeming the issue to be important: for example, their value system might not deem a specific problem worthy of attention, or they might be generally less oriented toward the environment (Newig et al. 2018; Tauro et al. 2018). Two questions are of interest: can trends be discerned in how knowledge of an environmental issue and interest in it develop? And, is understanding such trends helpful for engaging people in the management of the issue?

To explore the foregoing questions, in this article we consider invasive alien species in the Netherlands and examine the development of scientific and public attention for them and available insights into them. Ecologists contend that invasive alien species are a major threat to biodiversity; such species give rise to annual costs estimated to range from €12 billion for the EU (Shine et al. 2010) to €120 billion for the USA (Pimentel et al. 2005). Dealing successfully with invasive alien species is increasingly recognized as requiring the involvement of local communities (Niemiec et al. 2016; Stokes et al. 2006; Verbrugge et al. 2013). Perceptions of invasive alien species can change, however, affecting the likelihood that the general public will become involved in managing a particular species. For example, in Britain and Ireland, *Rhododendron ponticum* has changed from being regarded as an exclusive garden plant to being seen as a costly invader (Dehnen-Schmutz and Williamson 2006). In South Africa, prickly pear (*Opuntia ficus-indica*) was initially seen as an important fodder crop, but is now regarded as hampering livestock productivity (Shackleton et al. 2019).

The acquisition of additional knowledge plays an important role in raising the public’s attention for an invasive alien species. This article aims to discern trends in that dynamic. To do so, we examine the development of public and private salience regarding invasive alien species, as this determines the problem status of a species. We consider salience to be measurable in terms of attention paid to a species and the understanding of that species. In the “Analytical Elements” section, we elaborate on the concepts of salience, and how changes in salience can be thought of as trajectories across problem statuses. In the “Methodology” section, we explain how we reconstructed the development of scientific and public salience for thirteen invasive alien species in the Netherlands, based on numbers of publications in newspapers and on scientific articles. We discuss the species selected, the query used to search for publications, and our coding. In the “Results” section, we start by presenting the problem status per species, followed by the trajectories across these statuses. From these reconstructions, we derive how scientific and public salience develop from a latent problem status toward a manifest problem status. We then identify three species representing the different trajectories, and for these we analyze the content of the newspaper and scientific articles. We code for action and inertia, and how that changes along the problem status trajectories. Our approach serves to show the merit of looking at invasive alien species through the lens of problem status, and gives us an idea of whether a species’ problem status has added value for achieving the involvement of a community. We end with a conclusion, some reflections on the article, and suggestions for future research.

## Analytical Elements

In this section, we outline three analytical elements this article is built on: the salience of an issue to the public and science, the development of salience, and action undertaken or inertia occurring regarding an invasive alien species. In the “Methodology” section we link these to three research steps.

### Scientific and Public Salience

**Fig. 1:**
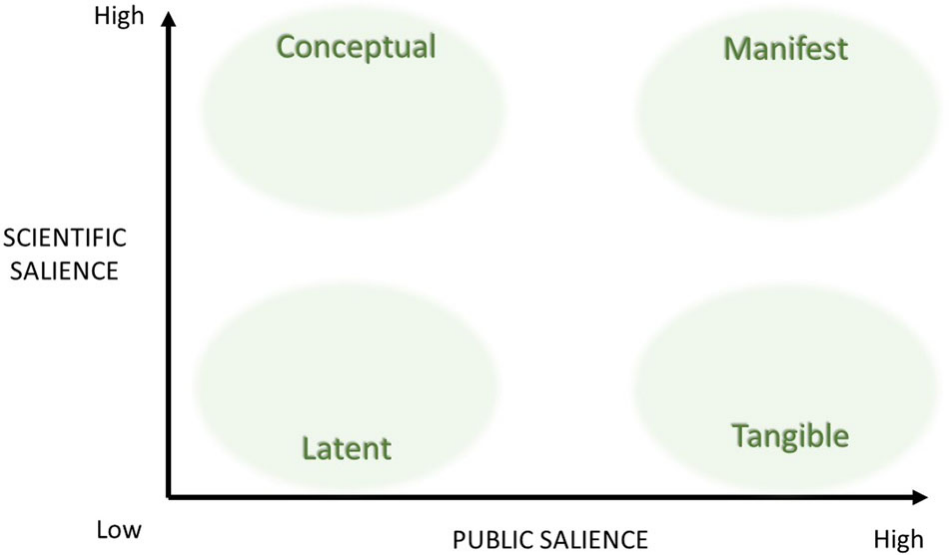
Problem statuses defined by two dimensions: salience to the public on the horizontal axis, and scientific salience (within all disciplines) on the vertical axis

We were interested in the combination of attention paid to a species and the understanding of that species, which together we call “salience”. This combination is inspired by distinctions made earlier between certainty of science and certainty of values, by e.g., de Boer et al. (2010), Hurlbert and Gupta (2015), Van Enst et al. (2014), and Gormley (1986). The vertical axis of Fig. 1 represents the scientific salience of an invasive alien species, meaning the amount of attention accorded to it by scientists and also scientists’ understanding of that species. This measure is mostly quantitative and does not say much about the quality of the knowledge available. Likewise, public salience on the horizontal axis is a measure of the amount of public attention accorded to a species and also the public’s understanding of a species; it does not say anything about whether that attention is warranted or not. By “public” we mean both the general public and government, as is further operationalized in the “Methodology” section. Both variables are intended to be generalizations, but obviously there are differences within the two groups. Scientists do not necessarily agree on how to deal with alien species (e.g., Boltovskoy et al. 2018; Simberloff et al. 2012; Valéry et al. 2009), nor do community members (see e.g., Epanchin Niell 2010; Graham and Rogers 2017; Klepeis 2009). Since we aim to derive general trends in the development of both variables, in this article we will not explore such differences.

The two variables result in four problem statuses, which are depicted in Fig. 1.

We define these problem statuses as follows: a conceptual problem is one for which ample scientific knowledge is available for predicting, which impacts will occur where, thereby allowing a technocratic type of management. Support from a community or government for such measures may, however, be lacking, since conceptual problems as we define them, are characteristically perceived as having little relevance for society. Conversely, a tangible problem is very salient to the public. However, low scientific salience makes informed management difficult to achieve, and therefore tangible problems can be expected to encourage perception-based management. When scientific knowledge is absent and the problem has low public salience, we are dealing with a latent problem. Since there is no public or scientific concern to act on, latent problems typically do not stimulate any management. Manifest problems are the complete opposite: similar to conceptual problems, they are characterized by a good understanding of which impacts will occur when, and are compounded by great public concern. Note that these statuses are not inherent to a species but change through time and between contexts. A species with latent problem status may acquire manifest problem status in a different region or 10 years later; we will elaborate in the next section.

In this article we apply the foregoing typology to invasive alien species, but the typology could be applied to other environmental problems, such as groundwater contamination, the extinction of a species, and sea-level rise (Vaas et al. 2020). The next section presents three hypothetical trajectories along which the problem statuses can change, which is this article’s second analytical element.

### Problem Status Trajectories

Changes in opinions about invasive alien species occur frequently. They could result from changes in distribution: a species introduced as a garden ornamental may cause great economic losses to farmers if it invades pasture (Vaz et al. 2017). But people’s perceptions may also change over time due to changes in what they value, or insights they have acquired (Shackleton et al. 2019). Many alien species were considered beneficial when they were introduced, such as the rainbow trout that was introduced in South Africa for fishing, or, as in the case of the cane toad in Australia, were intended to combat another pest (Caplat and Coutts 2011). If they, subsequently, also turn out to have major negative impacts, their management may become urgent. In other instances, a species initially perceived as a major pest that is zealously managed turns out to have some favorable characteristics (Davis et al. 2011). This was the case with tamarisk shrubs in the USA, which were believed to deplete groundwater and so were heavily suppressed from the 1930s onwards, which cost US$80 million between 2005 and 2009 alone. However, their water consumption turns out to be comparable to that of native counterparts, and they are the preferred nesting habitat of the endangered native willow flycatcher (Davis et al. 2011). In sum, changes occur in perceptions of the problem a species poses, both from a scientific and a societal perspective.

**Fig. 2:**
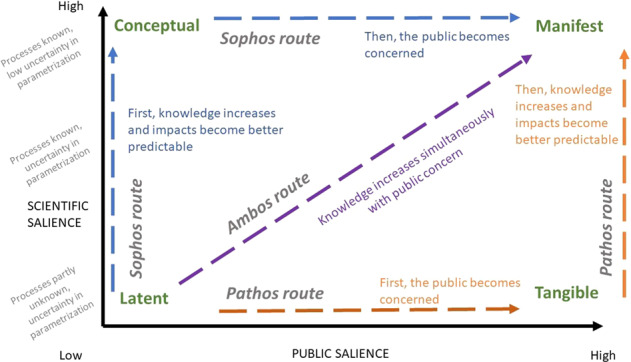
Three hypothetical problem status trajectories: *Sophos*, *Pathos*, and *Ambos*

Whereas changes in perceptions are well researched, studies have paid scant attention to how scientific and public perceptions change relative to each other. Looking at our grid of problem statuses, one can imagine that a species that initially has latent status will progress toward a conceptual status as additional knowledge becomes available, and then will appear on the radar of society and be pushed toward a manifest status. Alternatively, concern might first emerge among the public, which would assign a species a tangible status, after which scientific efforts to understand the species could result in the species attaining manifest status. In another conceivable trajectory, both scientific and public salience would increase simultaneously. Figure 2 depicts these three hypothetical itineraries, which we have called *Sophos*, *Pathos*, and *Ambos*^1^, respectively.

In this article we reconstruct the trajectories across problem statuses for thirteen invasive alien species in the Netherlands, to see whether there is evidence for the existence in practice of the trajectories shown in Fig. 2. We will examine whether the public only become interested once there is ample scientific insight into an invasive alien species, or whether scientific attention follows behind public concern. To illustrate the merit of looking at a species’ problem status, we conduct a small experiment by zooming in on the salience trajectories of three invasive alien species. We explore whether problem statuses of invasive alien species can be linked to society’s inertia or action regarding that species.

### Inertia or Action?

An earlier paper showed how latency results in difficulty in identifying stakeholders: when information on impacts of an invasive alien species is lacking, people cannot form an opinion regarding that species (Vaas et al. 2019). After more information on an invasive alien species becomes available, people can articulate opinions about the species, and stakeholder groups emerge. A community will then also be more likely to act in response to a species, since they have a stake involved. Following this reasoning, a conceptual, manifest, or tangible problem status should be conducive to action: there are clear stakes involved and these stakes affect a community.

Looking at the literature on species invasion, however, it is clear that the opposite situation, one in which many stakes are involved and plenty of scientific knowledge is available—what we refer to as a manifest problem—can also result in inertia. For example, cacti in South Africa cause major harm to biodiversity and human health, but also serve an agricultural and ornamental purpose. The discrepancies hamper collaboration and cohesion between stakeholders, which in turn limits the development and implementation of management strategies (Caplat and Coutts 2011; Novoa et al. 2016). A “conflict species” is one for which the costs and benefits involved are distributed across multiple actors (Novoa et al. 2018). Dealing with multiple stakes and stakeholders in order to manage such species is where invasive species management becomes particularly tricky (Woodford et al. 2016). In such cases, the aspiration of eradication becomes unattainable. For example, pine trees (*Pinus* species) planted in the 1930s in South Africa to provide timber are now invading the native fynbos shrubland. Foresters and conservationists have opposing stakes, and while the trees continue to spread, inertia continues as the conflict becomes increasingly intractable. Moreover, when stakes are aligned, the amount of resources required can result in a deliberate decision not to instigate action against an invasive species. The increasing scale and rate of spread of invasives in the Anthropocene have made living with an invasive a tenable course of action too (see, e.g., Head et al. 2015).

Like high public salience, high scientific salience, i.e., the availability of ample knowledge, can have adverse effects (Cortner 2000; Van Enst 2018). The case of the zebra mussel shows how knowledge availability is itself not enough: despite the large body of literature available on this alien species, nature managers were unprepared for dealing with it (Byers et al. 2002). Both policy makers and scientists can use knowledge strategically for their own aims. Policy makers may ignore insights that run counter to their preferences, or may use uncertainty and contradictory facts as justifications for postponing decision-making. The knowledge produced by scientists may have been generated by ignoring topics or stakeholder positions that the scientists deemed irrelevant (Turnhout et al. 2007; Van Enst et al. 2014). Thus, greater public and scientific salience cannot only invoke action but may also result in inertia.

How salience results in action is outside the scope of this article. Models such as “reasoned action theory” (Fishbein and Ajzen 1975), “planned behavior theory” (Ajzen 1991), and the “integrated behavioral model” (Kasprzyk et al. 2018), would lead one to argue that salience of an issue changes an individual’s attitude or the social influence exerted on that individual through norms. However, we do not aim to find causal explanations but merely to explore correlation. Moreover, we are interested in action on the part of a community or government as a collective entity, which is one level of abstraction higher.

Below, the three analytical elements presented above will be linked to three methodological steps.

## Methodology

In this article we assess the problem statuses of a carefully selected set of invasive alien species, and reconstruct the development of public and scientific salience that led to that particular problem status. We illustrate the usefulness of the problem status lens by zooming in on three invasive alien species. The three research steps for doing so are described below, as is the selection of species focused on.

### Thirteen Invasive Alien Species in the Netherlands

We looked at the invasive alien species present in the Netherlands, which is a spatial scale at which a comprehensive impression of action on the part of the community and government can be obtained without having to account for differences in jurisdiction. The Dutch species database lists 148 invasive alien species in the Netherlands (Nederlands Soortenregister, accessed February 19, 2019), from which we selected thirteen species. These are the species mentioned in publications of more than one of the four sources we considered to be representative of four different actor groups: the scientific community, civil society, nature management organizations, and government. We opted for these four diverse sources in order to prevent a bias toward scientific or public salience. The scientific source we selected was the article by Verbrugge et al. (2013), in which a group of invasive alien species that have different levels of appeal and impact on biodiversity in the Netherlands is presented. As a governmental source, we used the species listed by the EU Regulation on Invasive Alien Species to be managed by the Member States (European Parliament, Council of the European Union 2014). Our civil society source was the information on the most important invasive alien species in the Netherlands provided by the chairman of Platform Stop Invasieve Exoten (platform stop invasive aliens, the main civil society organization in the Netherlands concerned with invasive alien species) in response to our request (Reinhold pers. comm. 2019). As representative source for nature management organizations, we used the article by Holtjer (2009) in Boomblad (a magazine published by Alterra, the current Wageningen Environmental Research institute linked to Wageningen University), which mentions various invasive species. To mark the national government’s establishment of its first Team Invasieve Exoten (Invasive Aliens Team), Boomblad published a list of the most prominent alien species in the Netherlands compiled by several nature management organizations. We list the species mentioned by these different sources in the online resource accompanying this article and have highlighted the species mentioned in more than one source. As newspaper articles often conflate coypu (*Myocastor coypus*) and muskrat (*Ondatra zibethicus)*, we excluded muskrat. We added two species: Japanese knotweed *(Fallopia japonica)*, because it has attracted much attention recently (LexisNexis shows multiple hits per day), and quagga mussel *(Dreissena bugensis)*, because our civil society source mentioned that this is a species for which scientific salience has changed, which happens rarely. Table 1 shows the final result: a list of thirteen species for which we reconstructed the problem status trajectories, as explained in the next section.

**Table 1:** The thirteen species for which we reconstructed the problem status trajectories.

Taxonomic group	Common international name	Common Dutch name	Scientific name	Source
Invertebrate	Asian tiger mosquito	Aziatische tijgermug	*Aedes albopictus*	CS, S
Mammal	Coypu	Beverrat	*Myocastor coypus*	G, NMO
Bird	Egyptian goose	Nijlgans	*Alopochen aegyptiacus*	G, NMO
Aquatic plant	Floating pennywort	Grote waternavel	*Hydrocotyle ranunculoides*	NMO, S
Mammal	Gray squirrel	Grijze eekhoorn	*Sciurus carolinensis*	NMO, S
Bird	Indian crow	Japanse huiskraai	*Corvus splendens*	CS, S
Terrestrial plant	Japanese knotweed	Japanse duizendknoop	*Polygonum cuspidatum*	CS
Mammal	Pallas’s squirrel	Pallas eekhoorn	*Callosciurus erythraeus*	G, NMO
Invertebrate	Pumpkinseed sunfish	Zonnebaars	*Lepomis gibbosus*	NMO, S
Freshwater invertebrate	Quagga mussel	Quaggamossel	*Dreissena bugensis*	CS
Mammal	Raccoon	Wasbeer	*Procyon lotor*	G, NMO
Freshwater invertebrate	Red swamp crayfish	Amerikaanse rivierkreeft	*Procambarus clarkii*	G, NMO, S
Bird	Ring necked parakeet	Halsband parkiet	*Psittacula krameri*	CS, NMO, S

The source indicates where the species was listed as important. The species names are as given in these sources

*G* governmental source, *CS* civil society source, *NMO* nature management organization, *S* scientific source

### Reconstructing Trajectories

During the first research step we established the current problem status in the Netherlands for each of the thirteen species. The second step was to reconstruct the trajectories toward these statuses. This reconstruction enabled us to verify whether the hypothetical trajectories depicted in Fig. 2 do indeed occur in practice, and to select a representative species per trajectory for which we could assess the occurrence of inertia and action.

We defined the problem status of a species by its public and scientific salience. As a proxy for public salience, we took the total number of publications in Dutch newspapers and magazines in which that species was mentioned. A problem that raises concern among a community because it poses a real or perceived threat to their livelihoods can be expected to receive attention from journalists. We therefore searched the records in the LexisNexis database for mentions of the scientific names and common Dutch names as listed in Table 1. The publications in LexisNexis date from 1990 onwards, but the earliest record for any of our thirteen species was from 1997. For scientific salience we examined the records in Elsevier’s Scopus database for all disciplines, which has records on research articles published from 1980 onwards. The number of articles referring to a particular species can be considered a proxy for the scientific attention that has been given to that species. We assumed that the greater the number of scientific publications, the more reliable the predictions of a species’ impacts will be, due to better understanding of processes and higher parametrization. In the Scopus database, we searched only for scientific names, as adding the common names made little difference in number of hits. Some species have several common names, others are mainly referred to by their scientific name, and we wanted to prevent this from confounding the number of hits. For each of the thirteen species, we downloaded an overview of the number of publications from the Scopus and LexisNexis databases. Included were all publications recorded from 1980 in Scopus and from 1997 in LexisNexis. Our search for Scopus publications on the Asian tiger mosquito resulted in the notification “the number of publications is too large to process” without further specification, and so we added “alien OR nonnative OR invasive” to the query. We deem this exception admissible, given that the resulting number of publications still exceeded that of the other species. Its characterization as a manifest species is therefore justifiable.

To establish the problem status per species, we compared the number of records in Scopus and LexisNexis across the species. The four species with the most LexisNexis records were ranked highest for public salience. The four species with the most Scopus records were ranked highest for scientific salience. Conversely, the four species with the fewest LexisNexis records were ranked lowest for public salience and the four species with the fewest Scopus records were ranked lowest for scientific salience. If a number did not fall within the four highest or four lowest numbers of publications, it was ranked as intermediate. Combining the rankings on these two indicators determined a species’ location in the salience grid.

To reconstruct the trajectories of the species, we examined the sequence in which both indicators developed, starting in 1997, which is the earliest LexisNexis record. If the number of Scopus records increased before the number of LexisNexis records increased, we deemed the species to be following the *Sophos* route from Fig. 2. For a species to be assigned to the *Pathos* route, the LexisNexis records had to increase before the Scopus records; if both sources increased concurrently, the assigned route was *Ambos*. These reconstructions revealed which routes exist in practice. Whether it is worth understanding these routes was explored by focusing on three species, as described below.

### Illustrating Trajectories in Practice: Action and Inertia

What stimulated our research was the challenge of engaging and involving communities in the management of invasive alien species. We think that the challenge could be diminished by assessing the problem status of a species; to and we illustrate this, we zoomed in on a small subset of species to ascertain whether any trends emerge when the action or inaction regarding these species is viewed from the perspective of their problem statuses.

Our reconstructions of the trajectories enabled us to derive a few archetypical trajectories. For each of these archetypical trajectories, we selected one representative species and then assessed the occurrence of action and inertia. The indicators for our assessment are listed in Table 2: they vary between the content and the quantity of publications. We took three species, each with a different problem status. Distinguishing between governmental and community action or inertia, we then examined action undertaken and inertia within the Netherlands regarding these species. Responsibility for invasive alien species management is divided across multiple levels, from the European Union all the way down to water authorities and municipalities (European Parliament, Council of the European Union 2014; Provincie Gelderland 2018). In addition to these de jure institutions, de facto institutions often emerge to complement the government policies (Sullivan et al. 2017). If community actors feel that government policy is not sufficient, they may develop their own initiatives, as local Landcare groups have done for the invasive weed serrated tussock *(Nassella trichotoma)* in southeastern Australia (Marshall et al. 2016).

**Table 2:** The databases searched (left column) to establish problem statuses, inertia, and action (right column) for different species, based on either number or content of the records (middle column)

Source and search query	Assessment indicators	Indicator of:
LexisNexis www.academic.lexisnexis.nl LexisNexis>Power search>search terms [scientific name and common name] and select source by type: Dutch news	Number of records	Public salience
	NVivo analysis of content	Community action; community inertia; governmental inertia; governmental action
Official announcements of national government *zoek.officielebekendmakingen.nl*>*Parlementaire documenten* [scientific name and Dutch common name]	Number of records	Governmental action
	Scans of content	Governmental action
Open states archive for provinces *Openstateninformatie.nl* [abbreviated common name + scientific name]	Number of records	Governmental action
	Scans of content	Governmental action
Rivierenland water authority archive https://rivierenland.notubiz.nl/ [common name + scientific]	Number of records	Governmental action
	Scans of content	Governmental action
Scopus www.scopus.com ABS-TIT-KEY: [scientific name]	Number of records	Scientific salience

To establish governmental action, we looked at the records from several Dutch government bodies responsible for dealing with invasive alien species. The species listed in the Visserijwet (Fisheries Act) fall under the aegis of the Ministry of LNV (Agriculture, Nature, and Food Quality), while species listed in the Waterwet (Water Act) are under the aegis of the water authorities. The Ministry of LNV is also responsible for compliance with the European Regulation on Invasive Alien Species (European Parliament, Council of the European Union 2014), but has devolved implementation to the provinces. Thus, invasive alien species not explicitly assigned to other government bodies by Dutch law are the responsibility of the provinces. However, in the program presented by Utrecht province, tasks relating to alien invasive species have in turn been devolved to other agencies, among which are the water authorities. Doubts have been expressed regarding the appropriateness of the Ministry of LNV for dealing with the species, given its mandate, and it seems that Utrecht province is inclined to take measures itself (Provincie Utrecht 2019). Given these overlaps, for each species we looked at policy development by all three bodies. As it is not easy to acquire documentation of policy attention within a ministry, we combined records of regulations implemented and questions posed by the Second Chamber to Cabinet. The archives of the provinces and water authorities are all separate and differ in types of documents made accessible, which significantly constrained our analysis. Some provinces and water boards do not publish any of their meeting documents, and when they do, the documents are often scans that are not amenable to text searches. Thus, for the provinces we worked with the archive offered by the Open Staten NGO, which contain searchable documents of five provinces (Limburg, Flevoland, Utrecht, Zuid-Holland, and Noord-Holland). By researching the records of these five provinces, we could assume that each species would be invasive in at least one of the provinces and that documents would therefore be available. For the water authorities, we used the archives of the Rivierenland water authority. There were two reasons for this: the first is that according to an invasive alien species expert from the Water Authority Research Association (STOWA), Rivierenland water authority is proactive regarding invasive alien species (pers. comm., Van der Wal 2019). The second reason is that the Rivierenland archive is easily searchable.

We searched all three archives for the species chosen to represent archetypical trajectories, to assess the governmental action undertaken regarding these species. Although these archives enable us to gauge governmental action regarding an invasive alien species, it was not feasible to derive the absence of action (i.e., inertia) from them because the very existence of the records already testifies to some form of action having been taken by the government: at the very least, attention has been paid to the species. Therefore, to establish governmental inertia we used the same sources from which we derived community action and community inertia.

To assess governmental inertia, community action, and community inertia, we analyzed the 100 most recent LexisNexis publications per species. Using NVivo v.12 software, we coded for inertia (on the part of government and of the community), action (by government and by the community), and species’ impacts. We coded axially, setting the categories in advance and adjusting them during the process if deemed expedient (Wald et al. 2019). Community action took place when NGOs, individual citizens, or civil society organizations undertook some kind of activity regarding an invasive alien species. The activities ranged from organizing hikes, to lectures, to individual citizens undertaking management actions. Community inertia involved the same spectrum of actors but this time they were reported as not doing something or doing nothing (for example, failing to report a particular species). Governmental inertia was when a government body was reported not to be doing something regarding an invasive alien species (for example, not instigating management). We also coded for species’ impacts, negative and positive, to check for conspicuous differences in that characteristic. An overview of the databases searched per indicator is given in Table 2.

## Results

### Problem Status Trajectories

#### Problem Statuses per Species

The problem statuses of the thirteen selected species were assessed based on the number of scientific publications and Dutch news articles, as explained in the “Methodology”. The results are shown in Table 3. In the online resource of this article, per species the publications are set out across time; the species’ problem status trajectories will be discussed in the next section.

**Table 3:**
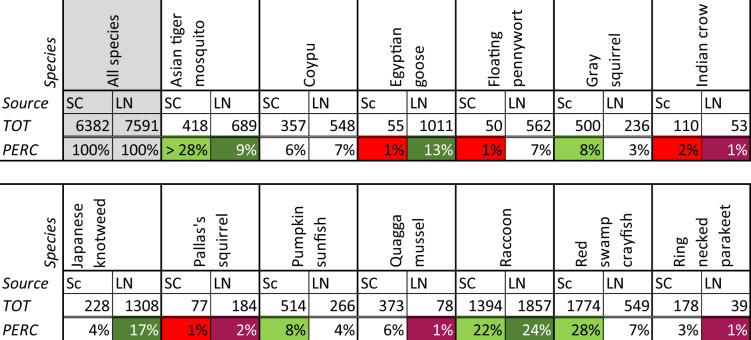
Relative numbers of Scopus (SC) and LexisNexis (LN) publications for thirteen invasive alien species in the Netherlands

The colors indicate the four lowest (red) and four highest (green) relative numbers of publications. For example: for the gray squirrel, the number of Scopus publications since 1980 is 8% of the number of Scopus publications for all thirteen species, which is why it is colored green. As explained in “Methodology”, there were many more records for the Asian tiger mosquito (28% refers to the refined query)

**Table 4:** The number of government publications for Asian tiger mosquito, broken down per source and year

Year of publication	Asian tiger mosquito																			Total number of publications
	2001	2002	2003	2004	2005	2006	2007	2008	2009	2010	2011	2012	2013	2014	2015	2016	2017	2018	2019	
Parliamentary						3	7	1	4	5		5	6	3	4	2	2	2		44
Provincial													1				1	2		4
Water authority																			1	1
All																				49

**Table 5:** Number of references (ref) per coding theme across newspaper articles (art) on Asian tiger mosquito

Code	Reference frequency	Content of references
Governmental action	80 ref. 56 art.	– Mostly eradication campaigns and monitoring activities by NVWA: placing traps, administering larvicides to standing water, and checking for breeding spots – Municipalities sometimes involved, to inform residents about the NVWA activities – A few references to research into the occurrence of the mosquito conducted by the European Centre for Disease Prevention and Control or the Dutch counterpart RIVM – Cooperation from the community required, as the NVWA needed to enter yards to check for mosquitos and potential breeding spots
Governmental inertia	28 ref. 28 art.	– Municipalities refer to the NVWA for management – Refraining from employing the army for eradication campaigns – Lack of mandate for government to create regulation regarding companies importing tires and bamboo plants – Pausing eradication of Asian mosquitos (*Ae. albopictus* and *Ae. Japonicus*) in the province of Flevoland while the NVWA and RIVM reassess strategies
Community action	37 ref. 27 art.	– Granting NVWA access to yards for eradication campaign – Reporting sightings of the mosquito – Removing potential breeding places for mosquitos in yards – NGO Platform Stop Invasieve Exoten calling for attention for the mosquito. Suing the national government, aiming for regulation regarding companies importing automobile tires – Researcher Bart Knols raising awareness about the mosquito and approaching the government; covenant between national government and tire-importing companies on installing traps and storing tires in a dry place – A company importing tires from countries where the mosquito has established stores these in a dry area and has mosquito traps installed around the area
Community inertia	3 ref. 2 art.	– People are not worried about the mosquito and take no measures – People do not check their caravans after returning from vacation in southern Europe
Negative impacts	66 ref. 56 art.	– Potential to spread diseases – Painful bite
Positive impacts	0	None
Ambiguous impacts	12 ref. 10 art.	– Uncertainties regarding future spread and source of exterminated mosquitos

*NVWA* Netherlands Food and Consumer Product Safety Authority, *RIVM* National Institute for Public Health and the Environment

**Table 6:** The number of government publications for Egyptian goose, broken down per source and year

Year of publication	Egyptian goose																			Total number of publications
	2001	2002	2003	2004	2005	2006	2007	2008	2009	2010	2011	2012	2013	2014	2015	2016	2017	2018	2019	
Parliamentary	1			3	1	1	1		2	3	10	3	3	6	7	1	1	1		44
Provincial								3	5		5	7	3	22	15	15	12	21		108
Water authority																1				1
All																				153

**Table 7:** Number of references (ref) per coding theme across newspaper articles (art) on Egyptian goose

	Reference frequency	Content of references
Governmental action	33 ref. 27 art.	– Monitoring the distribution of the species – The design of invasive alien species policies at provincial and municipal level – Provincial fauna management units hunting the goose – Shaking eggs or treating them with corn oil, typically at municipal level – Lifting hunting restrictions, for example in the province of Noord-Holland
Governmental inertia	4 ref. 4 art.	– City of Leeuwarden will not be undertaking action regarding the Egyptian goose, despite European regulations – Municipality of Rotterdam does not want to catch and kill the geese – Limited remuneration for damage caused by the geese
Community action	5 ref. 5 art.	– Public lecture on invasive alien species – Processing goose meat into food – Citizens reporting sightings of the species – Cooperation initiative at provincial level between governmental and nongovernmental actors
Community inertia	2 ref. 2 art.	– NGO Platform Stop Invasieve Exoten does not focus on the Egyptian goose since the government is already managing that species
Negative impacts	19 ref. 19 art.	– Threat to native species caused by breeding rapidly, taking over nesting sites of other birds, and killing chicks of other species – Can be aggressive toward humans – Posing a traffic hazard, since they are attracted by the grass between tram tracks – Like other geese: damage to crops and grasslands, and noise production
Positive impacts	3 ref. 3 art.	– Esthetic value – Food source for the European pine marten – Egyptian goose more favored than meadow birds as food for foxes
Ambiguous impacts	2 ref. 2 art.	– Since geese look for cover to breed while meadow birds prefer an open area, they may not pose a real threat – Having a new bird settle in the Netherlands is interesting, but might in the long term do damage

**Table 8:** The number of government publications for quagga mussel, broken down per source and year

Year of publication	Quagga mussel																			Total number of publications
	2001	2002	2003	2004	2005	2006	2007	2008	2009	2010	2011	2012	2013	2014	2015	2016	2017	2018	2019	
Parliamentary									1		1	1	1	10	9			1		24
Provincial															3		5			8
Water authority															5					5
All																				37

**Table 9:** Number of references (ref) per coding theme across newspaper articles (art) on quagga mussel

	Reference frequency	Content of references
Governmental action	26 ref. 20 art.	– Experiments using the mussel to filter water bodies. E.g., Brabantse delta water authority doing tests in a pond in Breda, and Amstel, Gooi and Vecht water authority constructing a “quagga filter” in the Sloterplas – Ministry of Economic Affairs stimulating experiments with the mussel to filter water – Monitoring of the distribution of the mussel, and research into the damage it can do, e.g., to sluices – EU regulation and plans regarding invasive alien species, among which the quagga mussel – 2017 International Maritime Organization Ballast Water Management Convention making treatment of ships’ ballast water obligatory
Governmental inertia	9 ref. 9 art.	– Water authorities have not responded to the appearance of the mussel for 5 years – Water authorities are struggling with the ambivalence about the impacts of the mussel
Community action	8 ref. 8 art.	– A dive center sinks Christmas trees to the bottom of the Reeuwijkse Plassen so quagga mussels will attach to them and improve visibility – Monitoring the presence of mussels in Amsterdam harbor or the province of Zeeland – Lectures and exhibitions on invasive alien species, among which the mussel
Community inertia	2 ref. 2 art.	– Arguing that the mussel is spreading too fast to contain
Negative impacts	31 ref. 27 art.	– Filtering activity reduces presence of plankton, negatively affecting other species – Reduction of fish means fewer sightings for divers – Clearer water increases plant growth because of more light infiltration – Adhering to surfaces such as electricity plant discharge pipes, boats, and docks – Outcompeting native mussel species, which has repercussions for native fish
Positive impacts	54 ref. 47 art.	– Filtering results in clearer water, which increases light availability – More available light boosts the growth of water plants and algae, which attracts birds and fish – Filtering is generally assumed to reduce the occurrence of cyanobacteria, benefiting swimming conditions for humans
Ambiguous impacts	13 ref. 9 art.	– The filtering capacity results in clear water, but also decreases the presence of plankton, which benefits some species but harms others – Does the mussel indeed decrease the presence of the cyanobacteria? – What will the long-term effects be?

**Fig. 3:**
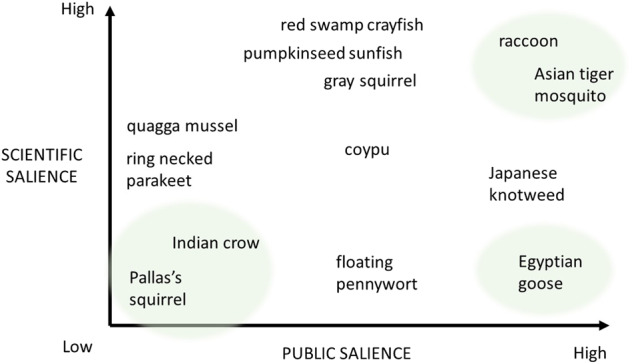
Problem statuses of thirteen invasive alien species in the Netherlands, as defined by their relative numbers of Scopus publications (vertical axis) and LexisNexis (horizontal axis) publications

There are two manifest species: the Asian tiger mosquito and raccoon both have the highest percentage of scientific and newspaper articles. There are also two latent species, namely Pallas’s squirrel and Indian crow; few scientific and newspaper articles mention these species. The coypu falls in the middle of all four quadrants, ranking medium for both scientific and newspaper publications. One species has tangible problem status: the Egyptian goose. It ranks low in terms of numbers of scientific publications but high in terms of number of newspaper publications. Somewhere between tangible and manifest problem status is Japanese knotweed, which ranks high on LexisNexis and intermediate on Scopus. In between latent and conceptual status are the ring necked parakeet and quagga mussel. Three species, the pumpkinseed sunfish, red swamp crayfish, and gray squirrel have a problem status between conceptual and manifest. A schematic overview is given in Fig. 3.

#### Problem Status Trajectory per Species

**Fig. 4:**
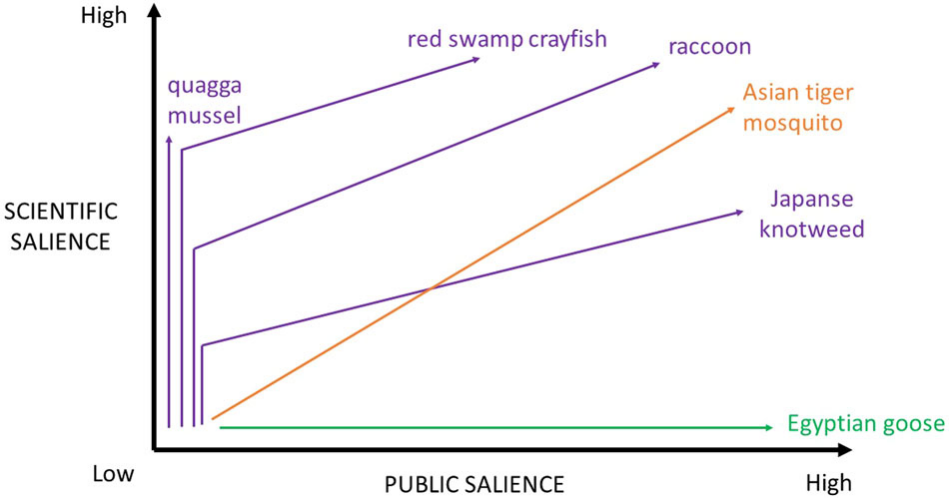
The three trajectories found in practice, indicated by different colors: purple for *Sophos*, orange for *Ambos*, green for *Pathos*. A trajectory represents the course of increasing public and scientific salience over time

**Fig. 5:**
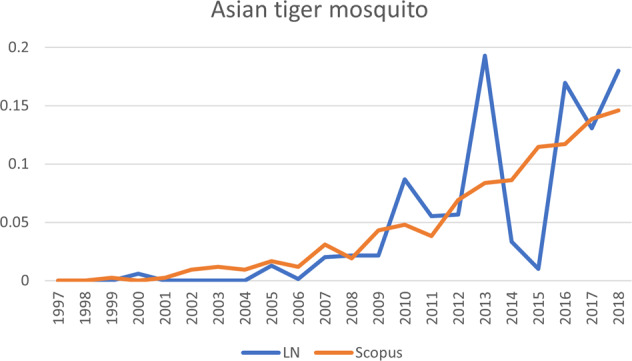
The development of publications for Asian tiger mosquito, one of the six invasive alien species shown in Fig. 4. Vertical axis: publications that mention the species, as percentage of total number of publications; horizontal axis: years of publication. LN LexisNexis, Scopus Scopus database

**Fig. 6:**
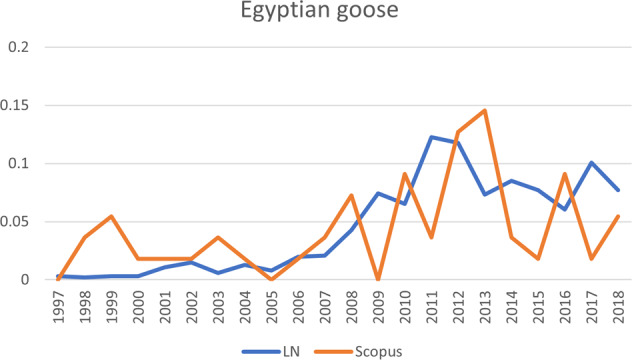
The development of publications for Egyptian goose, one of the six invasive alien species shown in Fig. 4. Vertical axis: publications that mention the species, as percentage of total number of publications; horizontal axis: years of publication. LN LexisNexis, Scopus Scopus database

**Fig. 7:**
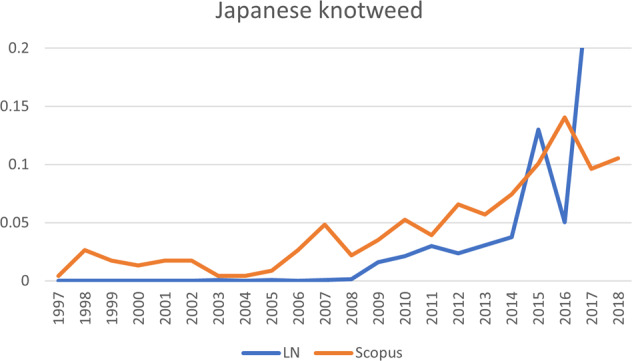
The development of publications for Japanese knotweed, one of the six invasive alien species shown in Fig. 4. Vertical axis: publications that mention the species, as percentage of total number of publications; horizontal axis: years of publication. LN LexisNexis, Scopus Scopus database

**Fig. 8:**
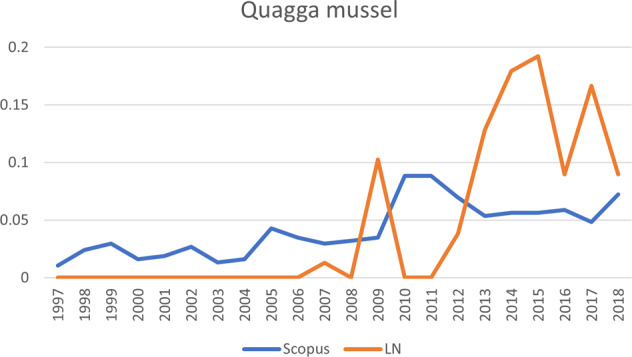
The development of publications for quagga mussel, one of the six invasive alien species shown in Fig. 4. Vertical axis: publications that mention the species, as percentage of total number of publications; horizontal axis: years of publication. LN LexisNexis, Scopus Scopus database

**Fig. 9:**
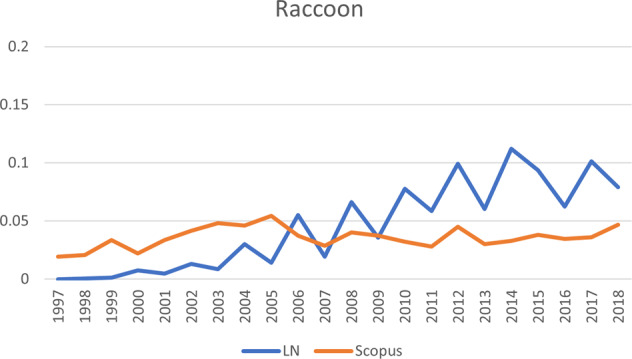
The development of publications for raccoon, one of the six invasive alien species shown in Fig. 4. Vertical axis: publications that mention the species, as percentage of total number of publications; horizontal axis: years of publication. LN LexisNexis, Scopus Scopus database

**Fig. 10:**
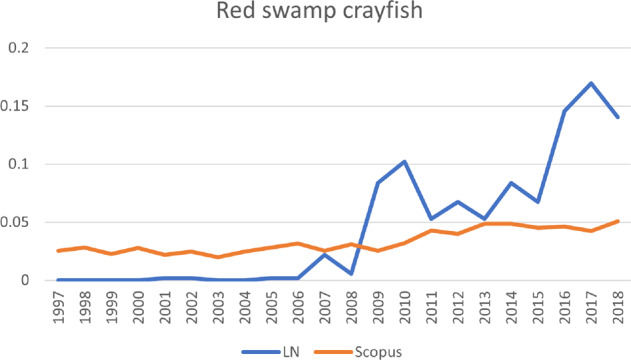
The development of publications for red swamp crayfish, one of the six invasive alien species shown in Fig. 4. Vertical axis: publications that mention the species, as percentage of total number of publications; horizontal axis: years of publication. LN LexisNexis, Scopus Scopus database

We derived the problem status trajectories of the species from the sequence in which scientific and public salience developed per species, as depicted in the graphs in the online resource of this article and Figs 5–10. The resulting problem status trajectories are shown below in Fig. 4, in which the colors indicating the three trajectories are the same as in Fig. 2. Note that we have only included species with a conspicuously high public or scientific salience, or with both high public and high scientific salience. Below, we discuss each trajectory, using an invasive alien species whose trend in attention typifies that trajectory.

Regarding the *Pathos* trajectory, it is important to note that the species following that trajectory (Egyptian goose) received conspicuously little scientific attention. The Egyptian goose has a tangible problem status, meaning that compared to the other species it attracted very little scientific attention: even though the graphs shown in Figs 5–10 do show some clear peaks for Scopus publications, the total number of scientific publications is much lower than for the other species. After 2008, the accelerating increase in public attention was followed by a slight increase in scientific attention.

Japanese knotweed, which also ranks very high in terms of public attention, ranks higher in terms of scientific attention than the Egyptian goose. However, when looking at the sequence of the scientific and public publications, the rate of scientific publications increased first and public publications did not follow until later. Therefore, Japanese knotweed follows the *Sophos* trajectory, even though overall it ranks higher in terms of public attention than in terms of scientific attention. A different pattern is shown by another species that followed the *Sophos* trajectory, namely the raccoon. For the raccoon, scientific attention grew first, and public attention followed, but it ranks highest in terms of both types of attention, rendering it a manifest problem. Public attention for this species also took off much later (around 2004), lagging behind a strong increase in scientific publications. Around 2007, public salience regarding Japanese knotweed followed the increase in scientific publications quite closely. For a third species following the *Sophos* trajectory, red swamp crayfish, public salience lagged even further behind scientific salience (around 2006). The differences between species in how far the increase of one type of salience lags behind another type of salience, are represented in Fig. 4 by the different points at which the lines downturn sharply. A fourth species that follows the *Sophos* trajectory is the quagga mussel. The scientific publications clearly increased before the newspaper articles and the overall number of newspaper articles remained very low, which is why this species ranks between a latent and conceptual species. For now, it is unclear whether public attention will catch up (in which case, the species will move toward a more manifest status), or whether the species will continue to attract mainly scientific attention.

The third trajectory, *Ambos*, is the trajectory followed by the other manifest species, the Asian tiger mosquito. Since scientific and public salience for this species started to increase at the same time (around 2006), this line runs diagonally across the grid.

## Problem Status Trajectories in Practice

Having derived three archetypical problem status trajectories, we will now look at representative species for each of them, to explore the action and conflict occurring per status. We zoom in on three species that are representative of the different trajectories found: the Asian tiger mosquito as representative of the *Ambos* trajectory; the quagga mussel representing the *Sophos* trajectory and the Egyptian goose as representative of the *Pathos* trajectory. For each of these species we will briefly discuss the governmental action and inertia, and community action and inertia as reported in the publications we analyzed, shown in Tables 4–9. We will then compare the three species to see if a link can be discerned between problem status, and inertia or action.

### Action and Conflict for Three Species

#### Asian Tiger Mosquito, *Aedes albopictus*

The first record of an Asian tiger mosquito in the Netherlands is from 2005. The species is thought to have traveled from Asia to Europe in automobile tires and bamboo plants. In 2017, 194 mosquitos were recorded, distributed over twelve municipalities; in 2018, there were 39 reports of the mosquito in the Netherlands (NVWA 2019). In June 2018, the European Center for Disease Prevention and Control deemed the mosquito to be “established” in the province of Limburg in the Netherlands (ECDC 2018), but in January 2019 its status was revised down to “introduced” (ECDC 2019). The mosquito is mainly feared for its potential to transmit the virus for diseases such as chikungunya, dengue, yellow fever, and zika; its bite is allegedly exceptionally painful. However, given the virtual absence of these viruses in the Netherlands, the national health institute RIVM assesses the health risks to be very low (Dagblad de 2018; Meershoek 2018; Van der Werff 2018). Nevertheless, whenever an Asian tiger mosquito is encountered somewhere in the country, the Netherlands Food and Consumer Product Safety Authority puts out traps and checks water sources in the environment, striving for complete eradication of the mosquito (Teunissen 2018).

##### Results from Government Databases

In the government documents, a focus on Asian tiger mosquito as a disease vector is very clear. The documents reflect how the species started out in 2006 as an occupational health hazard for employees in Dutch greenhouses cultivating bamboo, and evolved into a public health hazard upon its first sighting in the wild in 2010. The health risks are the reason for control, and when the mosquito’s alien status is mentioned, it is to reinforce the need to eradicate the species. Over a number of years, publications reveal disagreement about how to prevent more introductions of the species, with the LNV Ministry advocating covenants and parliament preferring import restrictions. In 2007, a covenant with bamboo-importing companies was signed; in 2009 it was made binding by converting it into a ministerial regulation. In 2011, parliament expressed criticism, arguing that bamboo imports should be prohibited entirely. There was similar disagreement regarding automobile tires: the covenant with tire importers signed in 2013 was also criticized by parliament for not being binding.

##### Results from Newspaper Articles

Asian tiger mosquito was the most densely coded species on almost every variable except for ambivalence regarding impacts: for ambivalence, it scored the same as the quagga mussel. The mosquito scored the highest of all species for both action and inertia, and there was an exceptionally large number of references for governmental inertia, most of which were about the government not deploying the army and refraining from imposing more restrictions on the companies importing bamboo or tires. Of the three species, the Asian tiger mosquito had the most references mentioning negative impacts, which might account for the large amount of action.

#### Egyptian Goose, *Alopochen aegyptiacus*

The Egyptian goose has been in the Netherlands much longer than the Asian tiger mosquito: the first record of breeding was in 1967, but the species had been introduced to Europe in the 18th century as an ornamental bird (Sanders-Kroeze 2018). According to the latest assessments, numbers of this species (which is in fact a duck) in the Netherlands in 2013–2015 ranged between 6900 and 11400 and the number seems to be stabilizing (RAVON 2019). Their harm lies in their aggressive behavior: they take over the territory and nests of other species, even of buzzards and goshawks. They can breed several times a year and even in mid-winter, but so far there are no indications that they have restricted populations of other water birds (SOVON). The species features in the EU Regulation of Invasive Alien Species under article 19, meaning Member States can decide whether to aim for eradication or control. Policy in the Netherlands is to contain the Egyptian goose: provinces develop policy for this and then typically grant a mandate to the Fauna Control Unit (Fauna Beheer Eenheid), as Utrecht and Gelderland provinces have done (Provincie Gelderland 2018; Provincie Utrecht 2019).

##### Results from Government Database

This species has the highest number of government records. Unlike the Asian tiger mosquito, it mainly features in provincial documents rather than in national documents. In common with other alien species, its alien status is used to emphasize the need for management but is not the main reason why it should be managed—that reason is the danger the geese pose to air traffic around Schiphol airport. There are therefore multiple covenants on limiting their presence. Thus, while the impacts of a native and an alien goose on air traffic are the same, the target number for the aliens population mentioned in these covenants is much lower than that of native species. This distinction is partly responsible for the dispute about this species, in which several NGOs and one party in Second Chamber have argued against specifically targeting alien species. The methods of eradication also spawned protest, and a court case revolved around the use of carbon dioxide to cull geese.

##### Results from Newspaper Articles

There is more mention of governmental action regarding the Egyptian goose than for the quagga mussel, but less than for Asian tiger mosquito. The Egyptian goose has the fewest references to governmental inertia, whereas for the other species such references reflect criticism of the government’s measures. Criticism relating to the Egyptian goose is mostly focused on action taken by the government. The goose has the lowest number of references for community action and community inertia, which might be because of the management measure applied (treating the eggs). There are only a few mentions of the impacts of the geese, so whereas in the case of the tiger mosquito its impacts could explain the salience, for the goose they do not. Nor can the reason for the goose’s high salience be readily derived from the publications analyzed.

#### Quagga Mussel, *Dreissena bugensis*

The quagga mussel originates from the Dnieper delta and Black Sea and it made its way to the Netherlands either after the construction of the Rhine–Main–Danube canal or via ships’ ballast water. In the early 2000s, the first quagga mussels were found in Hollandsch Diep and the Westeinderplassen, at densities of about 3000 quagga mussels per square meter. They have also been found in the canals in Amsterdam, and are often praised for cleaning the water and reducing the occurrence of the cyanobacteria and toxic algae that pose a danger to swimmers (Dorrestijn 2015; Tielemans 2015). As will be discussed below, these two impacts both give rise to some ambivalence about the species.

##### Results from Government Databases

The quagga mussel has the lowest number of records in government databases, and most of these express uncertainty or refer to ongoing research. As we will explain below, these documents reflect the predominantly positive impacts of the species: the mussel is referred to as improving the quality of swimming water by solving a recurring issue with cyanobacteria during summer. The alien status is only mentioned as an aside, as compounding the uncertainty of long-term impacts. It is not a reason for eradicating the species; rather, governmental actors appear to favor encouraging the mussel’s presence because of its supposedly positive impacts.

##### Results from Newspaper Articles

Despite this species having twice as many total references than the Egyptian goose, it has fewer references relating to action and inertia. Instead, most references pertain to impacts: positive, negative, and ambiguous. The filtering capacity of this mussel is most often presented as a positive impact, and therefore the action undertaken by the government and community most often exploits the mussel rather than attempting to contain it. As with the Asian tiger mosquito, human health appears to be the focus: most attention is on the quality of swimming water, rather than on repercussions on the ecosystem. A large proportion of the articles refer to pilot schemes in which the species is put to work. It being an alien is mentioned only sporadically, and then in conjunction with the uncertainties regarding this species.

### Comparing the Three Species

Below, we compare the three species regarding action undertaken and occurrence of inertia.

#### How do Problem Status and Action Undertaken Correlate?

The species with a manifest problem status, the Asian tiger mosquito, is also the species for which we found most activity undertaken by the government and the community: more than half of the articles contained a reference to such undertaken action. We found 37 references regarding community action targeting this species; this compares with five references for Egyptian goose and eight for quagga mussel. Some of the references to the mosquito concern people cooperating with the NVWA by granting access to their yard or reporting sightings, and covenants with companies importing tires or bamboo, but most pertain to the NGO Platform Stop Invasieve Exoten and scientist Bart Knols undertaking action. The low community action regarding the quagga mussel is in line with our expectations, but for the Egyptian goose we would expect the public salience to coincide with community action. However, although the citations are low in number, they do reflect structural community involvement. The bird collision covenants and the fauna management programs of the Fauna Control Units are both examples of long-term collaboration between government, nature management organizations, and private actors.

The largest number of references concerning governmental action again pertains to the Asian tiger mosquito. Given the impacts of the mosquito, the great amount of action is somewhat surprising, as the harm caused by this species is hypothetical: the mosquito might spread a certain virus if that virus were present in the Netherlands. Much less action has been undertaken for the quagga mussel, which has already changed the composition of entire lakes. The reason for the disparity in action undertaken becomes clearer when comparing the species’ problem statuses: Asian tiger mosquito has a manifest problem status, whereas quagga mussel has a conceptual problem status. The higher public salience of the Asian tiger mosquito could account for the larger amount of government action. The much smaller amount of governmental action regarding Egyptian goose appears at odds with this logic and is surprising, since that species has the highest number of records in the government databases. This difference might indicate that journalists are less interested in these activities than in the campaigns to exterminate the Asian tiger mosquito.

Overall, the problem statuses do partially coincide with action by the community and government, but other factors such as a species’ impacts might play a role as well.

#### How do Problem Status and Inertia Correlate?

Looking at the references for inertia compared to problem statuses, the highest number of references regarding inertia are found for species with a manifest status. The species with conceptual and tangible problem statuses have equal numbers of references regarding inertia. What do these references reflect: the degree of inertia, or the attention paid to actual or alleged inertia? Our analysis points toward the latter—that the number of references regarding inertia are more of an indication of the attention inertia attracts than to the existence of inertia itself. Thus, for the species with low public salience (quagga mussel), it could be expected that little attention would be paid to inertia, whereas it could be expected that there would be much attention for inertia in the case of the species with high public salience (Asian tiger mosquito and Egyptian goose). Asian tiger mosquito meets this expectation, but Egyptian goose does not. Two explanations come to mind: first, management of the Asian tiger mosquito has received much attention in general, and this included attention to measures not taken. The second possibility is that this species is framed as having a large potential impact on health, which has resulted in more scrutiny of the action undertaken. So here too, problem status can partially explain the occurrence of inertia, although another factor such as impact should not be ruled out.

## Discussion and Conclusion

In this article we have looked for trends in the development of understanding of and attention for invasive alien species on the part of the public and within the scientific community. We reconstructed the development of public and scientific salience for thirteen invasive alien species in the Netherlands and found three typical trajectories. The *Sophos* trajectory was followed by most species: it consists of scientific salience increasing first, followed sooner or later by an increase in public salience. The *Pathos* trajectory starts out with public salience predominating, whereas for the *Ambos* trajectory both public and scientific salience increase concomitantly.

Looking at invasive alien species and their problem status trajectories has added value in three ways. First, it offers a structured approach to analyzing the problem that a particular invasive alien species poses, which is rooted in a social dimension of invasive alien species. On this social dimension, red swamp crayfish and raccoon are the same but Egyptian goose is different. This finding is complementary to common ecological characterizations such as the barrier model (Richardson et al. 2000). Second, the problem status trajectories offer pointers for engaging communities in species management. For example, of the two species with a manifest problem status, one followed a S*ophos* and the other an *Ambos* trajectory, which suggests two things. First, that scientific salience is important for a species reaching a manifest problem status, either by taking the lead or going hand in hand with public salience. The importance of scientific salience might have to do with “alien” being an inherently science-based label. In the case of the Egyptian goose, we did indeed find that that label affected the management of the species. Ensuring scientific insights into a species would thus be an important contribution to promoting management of invasive alien species. However, and this is the second pointer problem status trajectories offer for engaging communities, public salience can precede scientific salience (although we found this for only one species, again the Egyptian goose). Less interest among the scientific community does not necessarily deter the public from according attention to the species but seems to be uncommon.

The third added value of problem status trajectories is understanding why a species is or is not the target of attention and action. In our illustration of these trajectories in practice by examining three invasive alien species in the Netherlands, we found that looking at the public and scientific salience of invasive alien species was helpful in understanding the action and inertia occurring regarding these species. Whereas Asian tiger mosquito as an invasive alien species is less impactful than the quagga mussel, it incites more action from both the government and the community. Knowing that the Asian tiger mosquito has a manifest problem status and the quagga mussel a conceptual status may not fully explain the different degrees of action, but it does indicate some dynamics at play. Likewise, why there are more references concerning inertia regarding the Asian tiger mosquito than there are for the Egyptian goose (which has been established in the Netherlands for much longer) and for the quagga mussel (before which management does not aim at containment) can be understood from the perspective of problem statuses.

Looking at the impacts of a species also yielded some insights. Potential positive impacts have resulted in the quagga mussel being enthusiastically exploited, whereas potential negative impacts have resulted in efforts to eradicate the Asian tiger mosquito and also in criticism of the action undertaken. The importance of impact has been pointed out earlier (e.g., Shackleton et al. 2019; Verbrugge et al. 2013) and it might be a second reason why scientific salience is important: to confirm the impacts of an invasive alien species. In such cases, ecological perspectives such as the barrier model (Richardson et al. 2000), which focus on the stage of invasion, might be less useful when the aim is to understand and promote action rather than a species’ impact on people.

An important limitation to this study is our use of number of records as a proxy for salience. As discussed by Byers et al. (2002), the existence of knowledge does not guarantee its dissemination and use, especially in the case of scientific articles behind a paywall. Moreover, articles can also be written for reasons other than the species’ invasiveness, such as a species’ added value to agriculture. We did not specifically search for invasion-related articles since we wanted to find action undertaken regarding a species regardless of whether that species is considered to be invasive. A final limitation to keep in mind is that whereas our study focuses on salience in the Netherlands, we used an international database of scientific articles. For a preliminary exploration of salience and problem status trajectories as conducted in this article, these limitations are acceptable. However, future research should pursue alternative methods for assessing salience.

Future research could also address three other aspects of the salience trajectories. The first aspect is differences in trajectories for different types of invasive alien species. People are known to respond very differently to invasive small mammals than to invasive plants (e.g., Remmele 2020). This might also affect the way salience develops for these species. The second aspect is whether there are set steps in a species’ progression toward increased public salience. What causes a species to move from having a latent status to having a tangible or manifest status? Knowing this would be helpful in engaging communities. The third aspect to address is to find causal links between problem status and action or inertia. Using publications to measure salience did not allow us to do that. Moreover, we looked at action at a national level, without looking into the reasons individuals did or did not engage in action. Differences within communities should not be overlooked (see e.g., Epanchin Niell 2010; Graham and Rogers 2017; Klepeis 2009), and engaging with models of individual behavior such as the Integrated behavioral model (Kasprzyk et al. 2018) would be helpful to that end.

We conclude that when looking from a higher level at promoting action regarding an invasive alien species, understanding a species’ problem status and the development of that species’ public and scientific salience has three advantages: it allows for a socially informed characterization of invasive alien species as a problem, enables understanding of the resulting action or inertia, and is helpful for engaging community involvement.

## Supplementary information

Supplementary Material

## Data Availability

Included in the Online Resource.

## Footnotes

1 The names are tongue-in-cheek references to emotion (the Ancient Greek *Pathos*), knowledgeable (the Ancient Greek *Sophos*), and combination (Spanish *Ambos*).
